# Temperature-Associated Variation in Evolutionary Rates of *FADS1* and *FADS2* in Rodents

**DOI:** 10.3390/ani16162600

**Published:** 2026-08-20

**Authors:** Chao Zhao, Zhao Liu, Xinglei Ding, Tian Xia, Shuo Dai, Guangshuai Liu, Jiaohui Fang, Honghai Zhang

**Affiliations:** 1 School of Life Sciences, Qufu Normal University, Qufu 273165, China; 2 Shandong Key Laboratory of Wetland Ecology and Biodiversity Conservation in the Lower Yellow River, Qufu 273165, China

**Keywords:** *FADS*, rodent, temperature, evolutionary rate, correlation

## Abstract

Environmental temperature is a major ecological factor shaping physiological and molecular evolution in mammals. Fatty acid desaturase genes (*FADS1* and *FADS2*) play essential roles in the biosynthesis of long-chain polyunsaturated fatty acids (PUFAs), which are critical for membrane structure and metabolic regulation. However, their evolutionary responses to climatic variation in wild mammals remain poorly understood. In this study, we analyzed 27 rodent species distributed across diverse climatic regions to investigate the relationship between environmental temperature and the evolutionary rates of the *FADS1* and *FADS2* genes. Phylogenetic comparative methods, including phylogenetic ANOVA, phylogenetic generalized least squares (PGLS), and branch model analyses, were applied to estimate ω (dN/dS) ratios and assess selection patterns. Branch-site model analyses were further performed to test for episodic positive selection acting on specific codons in low-temperature-associated lineages. Our results showed that species inhabiting lower-temperature environments exhibited significantly higher ω values for both *FADS* genes compared with those in warmer environments. PGLS analyses revealed consistent negative associations between temperature variables and evolutionary rates, particularly for BIO5, and branch model analyses further indicated elevated ω values in low-temperature-associated lineages relative to background branches. Branch-site analyses detected significant signatures of episodic positive selection in *Spermophilus dauricus* and *Microtus oregoni*, with candidate positively selected codons identified in both lineages. Similar temperature-associated evolutionary rate patterns were also observed in expanded mammalian datasets, suggesting a broader cross-taxa trend. Overall, these findings indicate that environmental temperature is associated with heterogeneous evolutionary rate patterns in *FADS* genes and suggest that lipid metabolism genes may be recurrently influenced by cold-related selective pressures across mammals.

## Simple Summary

Environmental temperature strongly influences how animals survive and adapt to their habitats. Fatty acid desaturase genes, particularly *FADS1* and *FADS2*, help animals produce important fatty acids that maintain cell membrane function and support energy metabolism. However, variation in the evolutionary rates and selective pressures acting on these genes in wild animals across thermal environments remains unclear. In this study, we examined 27 rodent species living in different climatic regions and compared the evolutionary rates of their *FADS1* and *FADS2* genes with environmental temperature data. We found that species living in colder environments generally showed higher evolutionary rates of these genes than species from warmer regions. Similar patterns were also observed when additional cold-associated mammals were included. These results suggest that temperature is associated with variation in the selective pressures on *FADS* genes and that such evolutionary variation may be related to differences in lipid metabolism among mammals inhabiting contrasting thermal environments. This study improves our understanding of how mammalian lipid metabolism genes evolve in relation to climatic temperature and provides a framework for investigating the responses of cold-associated species to ongoing climate change.

## 1. Introduction

Environmental temperature is a key abiotic factor that strongly influences the geographical distribution, physiological metabolism, and molecular evolution of animals [1,2,3]. Drastic temperature variations across spatial and temporal scales exert substantial natural selection pressures, driving the adaptive evolution of functional genes within species [4]. This phenomenon results in a broadly observed correlation between the evolutionary rates of specific genes and macroscopic climatic factors [5]. In particularly cold environments, endothermic animals face the significant challenge of preventing heat loss to maintain their core body temperature and ensuring cellular and systemic physiological homeostasis [6,7,8]. To endure extreme environmental stresses, mammals have developed multiple adaptive strategies, including membrane biophysical responses, energy metabolism reprogramming, and systemic physiological regulation [9,10]. Among these, the metabolic remodeling of polyunsaturated fatty acids (PUFAs) plays a crucial role [11].

The fluidity of the cell membrane serves as the physiological basis for transmembrane transport, receptor signaling, and membrane protein function [12,13]. Membrane lipid composition is sensitive to environmental temperature, and increasing the proportion of PUFAs can help maintain membrane fluidity under cold conditions, consistent with the homeoviscous adaptation hypothesis [9,14,15,16,17]. PUFAs are also involved in metabolic and thermogenic regulation, including pathways associated with brown adipose tissue, PPAR signaling, and non-shivering thermogenesis [18,19,20,21,22,23]. In addition, changes in PUFA composition have been associated with physiological tolerance to low body temperatures in hibernating mammals [24,25,26]. Together, these observations suggest that regulation of PUFA metabolism may represent an important component of mammalian responses to cold environments.

Fatty acid desaturases (FADSs) are essential enzymes that catalyze the introduction of double bonds into the carbon chains of fatty acids, playing a crucial role in the biosynthesis of long-chain PUFAs [27]. In the mammalian genome, the *FADS* gene family is typically organized into closely linked gene clusters, with each member exhibiting a high degree of homology and conservation in genomic structure, including exon/intron organization, suggesting that these genes originated from tandem gene duplication events early in vertebrate evolution [28,29,30]. *FADS2* encodes a Δ6-desaturase, whereas *FADS1* encodes a Δ5-desaturase [31]. Together with elongases from the ELOVL family, these enzymes coordinate the conversion of linoleic acid (LA) and α-linolenic acid (ALA) into long-chain PUFAs, ultimately producing long-chain products such as arachidonic acid (AA), eicosapentaenoic acid (EPA), and docosahexaenoic acid (DHA) [27,32]. These long-chain PUFAs serve not only as essential structural components of cellular membrane phospholipids but also as precursors for signaling molecules involved in inflammatory responses, neural development, and energy metabolism [33,34]. Given their critical role in catalyzing the biosynthesis of long-chain PUFAs, *FADS1* and *FADS2* represent important candidate genes for elucidating the evolutionary mechanisms underlying the adaptation of wild animals to extreme cold climates and energy metabolism [35,36].

Previous population genetic studies have demonstrated that the *FADS* gene cluster has undergone substantial selection in humans, particularly in populations experiencing distinct dietary and environmental conditions. Studies have reported selection signals in Greenlandic Inuit, Native American, and European populations undergoing major dietary transitions [36,37,38,39], indicating that *FADS* genes are evolutionarily responsive to ecological and nutritional pressures. However, most evidence has been derived from human populations, whereas the evolutionary patterns of *FADS1* and *FADS2* across wild mammalian species remain poorly understood. In particular, whether variation in environmental temperature is consistently associated with changes in selective pressures on these genes across independent mammalian lineages has not been systematically evaluated.

Rodents, the most species-diverse order of mammals, exhibit extensive ecological diversification across global climatic gradients. Their distribution ranges from tropical rainforests to high-latitude tundra, making them an ideal natural system for investigating thermal adaptation in endotherms [40]. In particular, rodent species with narrow geographic distributions and strong microhabitat dependence are less capable of tracking climate shifts through dispersal and therefore rely more heavily on in situ adaptation to cope with environmental change. As a result, their genomes often retain detectable signatures of climate-associated molecular evolution [41,42]. The present study examined 27 rodent species across a range of global climatic regions by integrating distribution data with environmental temperature datasets. We systematically evaluated the relationship between the evolutionary rates of the *FADS1* and *FADS2* genes and environmental temperature, and we further tested whether lineages exposed to extreme cold conditions exhibited signals of elevated evolutionary rates and potential lineage-specific adaptive responses. This study provides insights into the temperature-associated evolution of *FADS* genes in wild animals and offers a novel evolutionary genetic perspective for understanding the interaction between lipid metabolic genes and extreme climatic environments under natural selection pressures.

## 2. Materials and Methods

### 2.1. Species Sample and Climate Dataset

To reduce potential confounding effects from complex climatic adaptation signals in widely distributed species, we selected 27 rodent species with available whole-genome data and relatively restricted geographic distributions within defined zoogeographic regions. Occurrence records (latitude and longitude) were downloaded from the Global Biodiversity Information Facility (GBIF) [43], and erroneous or outlier records were carefully removed. Based on the cleaned geographic coordinates, three temperature-related bioclimatic variables—Annual Mean Temperature (BIO1), Max Temperature of Warmest Month (BIO5), and Min Temperature of Coldest Month (BIO6)—were extracted from the WorldClim database (https://www.worldclim.org/) at a 2.5 arc-min resolution. These climactic variables were selected to represent mean, upper-extreme, and lower-extreme thermal conditions, respectively, summarized across all occurrence points for each species using a capped-mean approach to reduce the influence of extreme values, and then averaged to obtain final species-level estimates. Subsequently, the 27 species were ranked according to the three climatic variables and further divided into three equally sized groups—Group 1 (Low), Group 2 (Medium), and Group 3 (High)—which were used in subsequent phylogenetic comparative analyses. We generated a distribution map based on the cleaned GBIF occurrence records to visualize the geographic distribution of the selected species and the spatial context of the climatic grouping. The 27 species were plotted according to their occurrence coordinates and classified into the corresponding climatic groups based on BIO5 values, providing a spatial representation of the macroclimatic differences among species included in the comparative analyses. Notably, the WorldClim variables used in this study represent broad-scale atmospheric conditions rather than the operative microclimates experienced by individual rodents. Therefore, species-level atmospheric temperature should be interpreted as a macroclimatic proxy rather than as a direct measure of physiological cold exposure.

### 2.2. Gene Sequence Acquisition and Phylogenetic Tree Construction

In this study, we first retrieved 15 coding sequences (CDSs) of the *FADS1* and *FADS2* genes from eight rodent species through the NCBI GenBank database (https://www.ncbi.nlm.nih.gov), which served as the initial seed sequences (Table S1). Subsequently, genomic data for additional species included in this study were downloaded from the NCBI to construct a local reference database. The seed sequences were used as queries, and BLASTN was employed to perform batch genome-wide homology searches within the local database to identify candidate *FADS1* and *FADS2* homologs. All candidate sequences were filtered using a stringent threshold (E-value < 1 × 10^−5^ and sequence identity > 70%) to minimize false-positive results. Detailed information on the genomes and scaffold-level annotations of newly identified genes is provided in Table S1. Multiple sequence alignment was performed using the ClustalW algorithm implemented in MEGA 11 [44], and the resulting alignments were manually inspected and refined to remove regions with missing data, ambiguous alignment, or indels, ensuring accurate codon-based alignment.

Phylogenetic reconstruction was conducted using a Bayesian inference (BI) approach. First, all *FADS* sequences were aligned using MAFFT in PhyloSuite with the auto strategy and codon alignment mode [45,46]. The best-fit substitution model (HKY + I + G) was selected using ModelFinder. Bayesian analysis was then performed using Markov Chain Monte Carlo (MCMC) sampling for 1,000,000 generations, with trees sampled every 1000 generations [47]. Convergence was assessed using the average standard deviation of split frequencies (ASDSF), with values below 0.01 indicating adequate convergence. Finally, the phylogenetic tree was visualized using the Interactive Tree of Life (iTOL) website [48].

### 2.3. Gene Evolutionary Rate Estimation and Comparative Phylogenetic Analysis

To evaluate the *FADS1* and *FADS2* evolutionary rates across species and their association with climatic factors, we estimated the ratio (ω) of nonsynonymous (dN) to synonymous (dS) substitution rates for each branch using the free-ratio model implemented in CODEML within the PAML package [49]. The one-ratio model, which assumes a single ω value across all branches, was used as the null model. Likelihood ratio tests (LRTs) were constructed by comparing the lnL between models to assess significance. The ω value serves as an indicator of the selection pressure acting on protein-coding genes: ω < 1 indicates purifying selection, ω = 1 suggests neutral evolution, and ω > 1 reflects positive selection. In this study, the ω values were used as the metric for measuring evolutionary rates, and all values were log-transformed to improve normality for statistical comparison and regression analyses.

A series of statistical and regression analyses was performed to examine the relationships between climatic factors and the *FADS1* and *FADS2* evolutionary rates. On the basis of the previously defined climatic categories for rodents, differences in ω values among groups were tested using phylogenetic analysis of variance (phyANOVA), followed by pairwise comparisons (*t*-tests) to identify specific group differences. When significant main effects were detected, the Holm correction was applied to adjust for multiple comparisons. All phyANOVAs were conducted in R using the ape, geiger, and phytools packages [50,51,52]. Subsequently, phylogenetic generalized least squares (PGLS) regression was used to evaluate potential correlations between climatic factors and the *FADS1* and *FADS2* evolutionary rates. These analyses were conducted using the caper package in R (version 4.2.3) [53]. The phylogenetic signal (λ) was estimated using the maximum likelihood (ML) method. A λ value close to 0 indicates weak phylogenetic dependence, whereas a value approaching 1 indicates a very strong phylogenetic signal. The overall model fit was evaluated using adjusted R-squared. All analyses were based on a time-scaled phylogenetic tree of the 27 rodent species [54]. To control for false positives arising from multiple testing across genes and climatic factors, all *p*-values from the PGLS analysis and phyANOVA were subjected to multiple testing with the Benjamini–Hochberg method, with a false discovery rate (FDR) threshold of <0.05 considered statistically significant.

### 2.4. Selection Pressure Analysis

We employed the branch model (two-ratio model), assigning Group 1, Group 2, and Group 3 as foreground branches, to verify whether low-temperature environments affect selection pressure on *FADS* genes. This approach allowed foreground and background branches to have different ω values, facilitating an examination of the selection pressure across different climatic groups. The one-ratio model served as the null model.

To further test whether the relationship between temperature and *FADS* evolution is generalizable across mammals, we expanded the dataset by including additional rodent species representing cold-associated or high-latitude lineages (*Pteromys volans*, *Microtus agrestis* [also referred to as *Euarvicola agrestis* in some recent taxonomic classifications], *Marmota marmota*, *Lemmus lemmus*, and *Chionomys nivalis*), as well as representative species from artiodactyls, carnivores and lagomorphs. Using these cold-adapted species as foreground branches, we repeated the branch model analyses described above.

We conducted branch-site model analyses to identify codons evolving under positive selection along predefined foreground branches to further investigate whether specific codons have undergone episodic positive selection within cold-associated lineages. Species belonging to the low-temperature group were individually designated as foreground branches, whereas the remaining species were treated as background branches. For each foreground lineage, the branch-site alternative model (Model A) was compared with the corresponding null model, in which the positively selected site class was constrained to neutral evolution (ω = 1), using likelihood ratio tests. For branches showing significant evidence of positive selection, Bayes Empirical Bayes (BEB) analysis was subsequently applied to identify candidate positively selected codons, with posterior probabilities >0.95 considered as supporting evidence.

## 3. Results

### 3.1. FADS Genes Identification and Climate Dataset Collection

**Figure 1:**
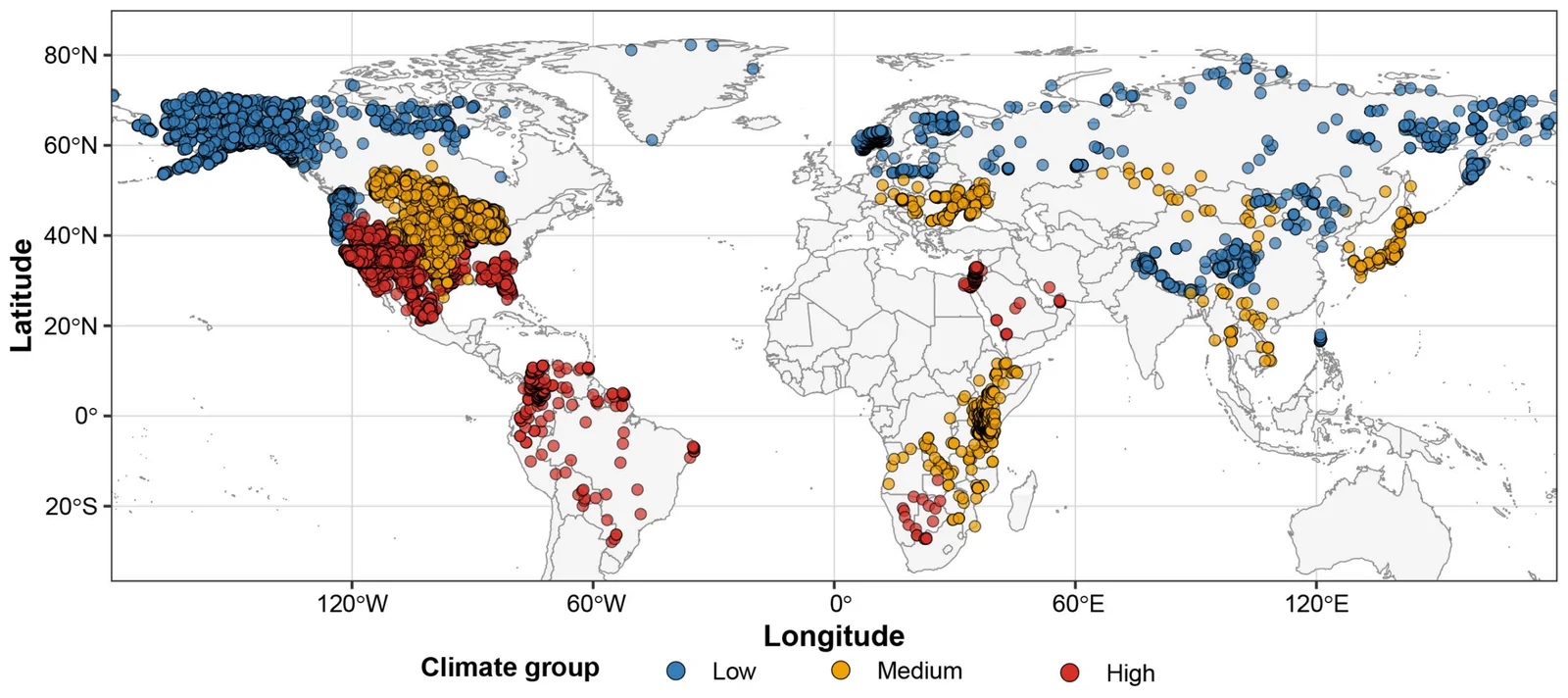
Geographic distribution and BIO5-based climatic grouping of the 27 rodent species included in this study. Species occurrence records were obtained from the Global Biodiversity Information Facility (GBIF), and climatic variables were extracted from the WorldClim database. The map was created using R with the packages sf and rnaturalearth.

We selected 27 rodent species from distinct geographic regions spanning tropical, temperate, and cold climatic zones to investigate the relationship between the evolutionary rates of *FADS* genes and temperature-related factors. Initially, annotated *FADS* gene sequences were retrieved from the GenBank database. For genes with incomplete or missing sequences, gene structures were re-identified using local BLASTN searches based on homologous sequences, resulting in a total of 52 valid gene sequences. For each species, geographic distribution records were combined with the following climatic variables extracted from the WorldClim database: BIO1 (Annual Mean Temperature), BIO5 (Max Temperature of Warmest Month), and BIO6 (Min Temperature of Coldest Month). The final species-level estimates are summarized in Table S2. The geographic distribution and BIO5-based climatic grouping of the analyzed species are shown in Figure 1.

### 3.2. Phylogenetic Tree of FADS and Calculation of ω Values

**Figure 2:**
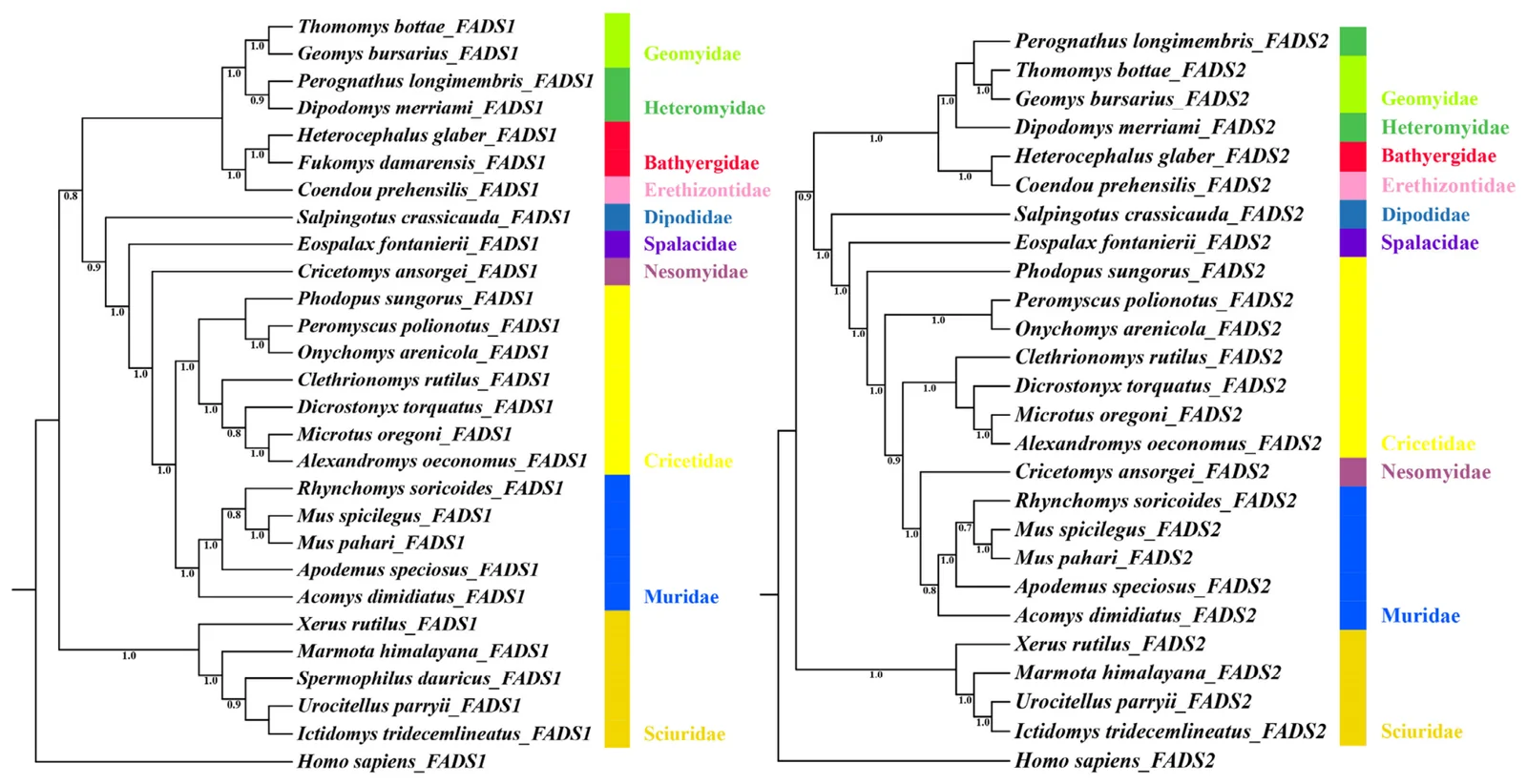
Bayesian phylogenetic trees of rodent *FADS1* (**left**) and *FADS2* (**right**).

Phylogenetic analyses of *FADS1* and *FADS2* were conducted separately, and both gene trees showed broadly consistent taxonomic clustering patterns. Within each gene tree, species generally clustered according to family-level relationships, with members of Muridae, Cricetidae, Spalacidae, and Nesomyidae forming closely related groups. Species from Heteromyidae and Geomyidae also clustered together, whereas Sciuridae, Erethizontidae, Bathyergidae, and Dipodidae formed distinct lineages (Figure 2). Most major nodes were supported by relatively high posterior probabilities, indicating that both *FADS1* and *FADS2* retain clear phylogenetic signals across rodents. Although some topological incongruences with published species-level phylogenies were observed, these differences may reflect gene-specific evolutionary histories or lineage-specific sequence variation [40,54,55]. Overall, the separate *FADS1* and *FADS2* phylogenies showed no obvious evidence of sequence misidentification or paralogous interference, supporting the reliability of the sequence datasets for subsequent analyses of evolutionary rates and climatic associations.

The ω values of the *FADS* genes were then estimated using branch models (free-ratio model versus one-ratio model) in PAML. The ω values obtained from the one-ratio model for *FADS1* and *FADS2* were 0.1037 and 0.0691, respectively, indicating that both genes are under strong purifying selection across rodents. This result is consistent with the conserved function of *FADS1* and *FADS2* as the rate-limiting enzymes in the synthesis pathway of long-chain polyunsaturated fatty acids, suggesting that the amino acid sequences responsible for maintaining basic enzymatic functions are subject to strict evolutionary constraints. The results of the likelihood ratio test (LRT) for the two genes showed that the free-ratio model fitted the data significantly better than the one-ratio model (*p* < 0.05), indicating that the evolutionary rates and selective pressures of the *FADS* genes have diverged significantly among different rodent species (Table S3).

The ω values of the terminal branches for each species, estimated by the free-ratio model, revealed that most species had ω < 1, indicating the action of strong purifying selection on the *FADS1* and *FADS2* genes in most rodents (Table S3). Notably, in the analysis of *FADS1*, the ω values for *Microtus oregoni* (2.7220) and *Spermophilus dauricus* (2.2063) were significantly greater than 1, indicating lineage-specific increases in nonsynonymous substitution rates and suggesting that these lineages may have experienced positive selection. In addition, *Clethrionomys rutilus* (0.5469) and *Alexandromys oeconomus* (0.8548) exhibited relatively elevated ω values, indicating a relaxation of purifying selection pressure. In the analysis of *FADS2*, *Urocitellus parryii* had an ω value of 1.1992, suggesting that positive selection may have occurred in this lineage. In summary, both *FADS1* and *FADS2* in rodents appear to be primarily subject to purifying selection. The ω value for the *FADS2* gene is generally lower than that for the *FADS1* gene, indicating that these genes may have experienced distinct patterns of selection pressure throughout rodent evolution. Notably, significant positive selection signals were identified in several branches, implying that adaptive evolution of the *FADS* genes may have occurred in these groups.

### 3.3. Statistical Comparisons of ω Values Among Climatic Groups

The 27 rodent species were classified into three groups based on three climatic variables to investigate the relationship between temperature and evolutionary rates. Phylogenetic analysis of variance (phyANOVA) and multiple comparison tests were then used to compare the ω values across the Low-, Medium-, and High-temperature groups. Overall, the results revealed significant differences in the ω values of the *FADS1* and *FADS2* genes among specific climatic groups.

**Figure 3:**
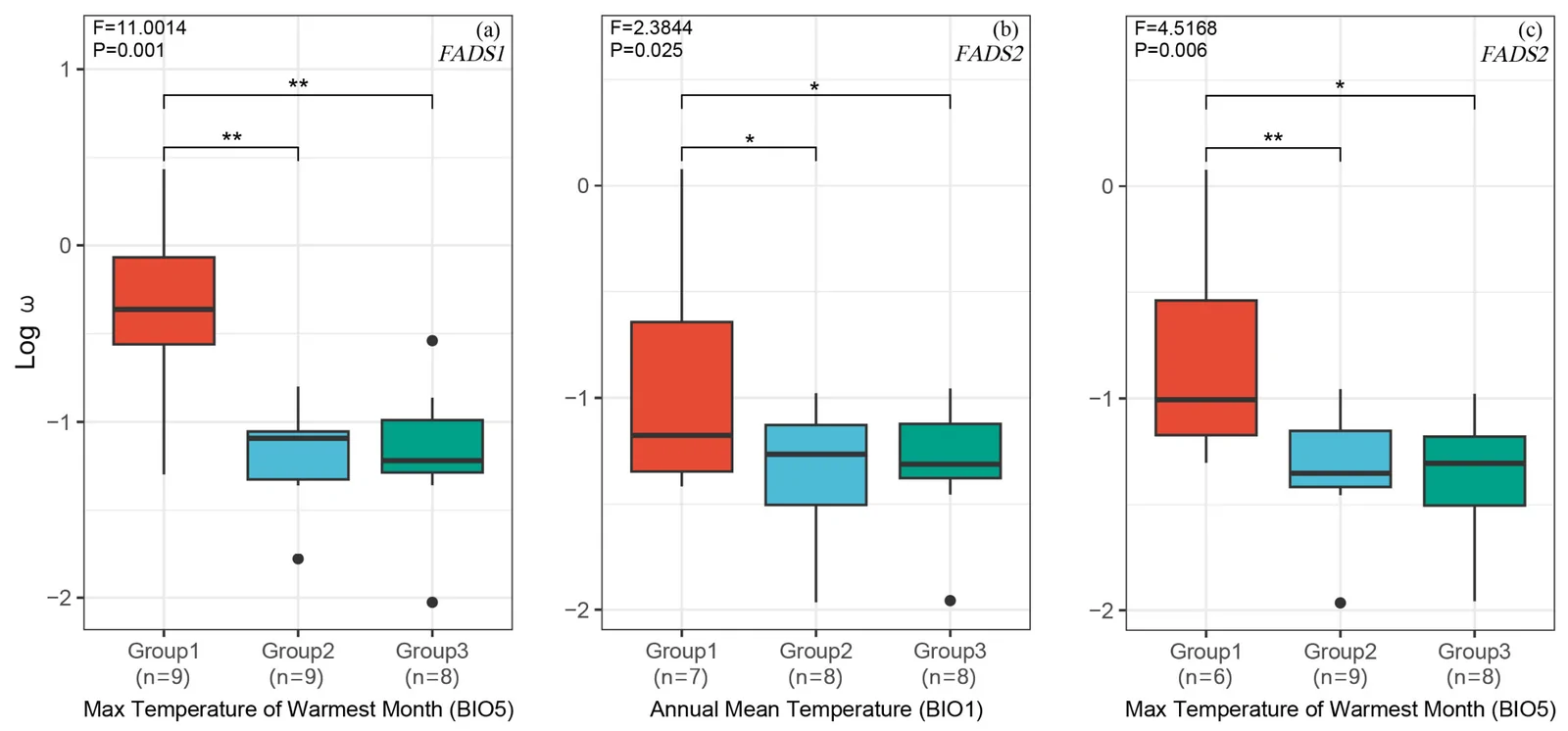
Statistical comparisons of ω values grouped by climatic factors. (**a**) Comparison of ω values of *FADS1* among three categories of BIO5. (**b**) Comparison of ω values of *FADS2* among three categories of BIO1. (**c**) Comparison of ω values of *FADS2* among three categories of BIO5. Species were classified into three climatic categories according to the rankings of the corresponding climatic factors. The F-value and *p*-value shown in the upper-left corner of each panel were obtained from the phyANOVA results. Pairwise comparisons were performed using *t*-tests; * and ** indicate statistical significance at *p* < 0.05 and *p* < 0.01, respectively.

For *FADS1*, a highly significant intergroup difference was observed among groups based on BIO5 (F-value = 11.0014; *p* = 0.001) (Figure 3a, Table S4). Post hoc multiple comparisons with Holm correction showed that the ω values of the Low-temperature group were significantly greater than those of both the Medium (*p* = 0.003) and High (*p* = 0.003) groups. However, no significant difference was detected between the Medium and High groups (*p* = 0.926). For *FADS2*, significant intergroup differences were detected among groups based on BIO1, BIO5 and BIO6 (BIO1: F-value = 2.3844; *p* = 0.025; BIO5: F-value = 4.5168; *p* = 0.006; BIO6: F-value = 2.3844; *p* = 0.025) (Figure 3b,c, Table S4). Post hoc tests further confirmed that the Low group exhibited significantly higher ω values than both the Medium- and High-temperature groups across these climatic factors. Collectively, these findings suggest that rodents inhabiting low-temperature environments generally possess higher evolutionary rates of *FADS* family genes.

### 3.4. Correlation Between FADS Evolutionary Rates and Climatic Factors

**Figure 4:**
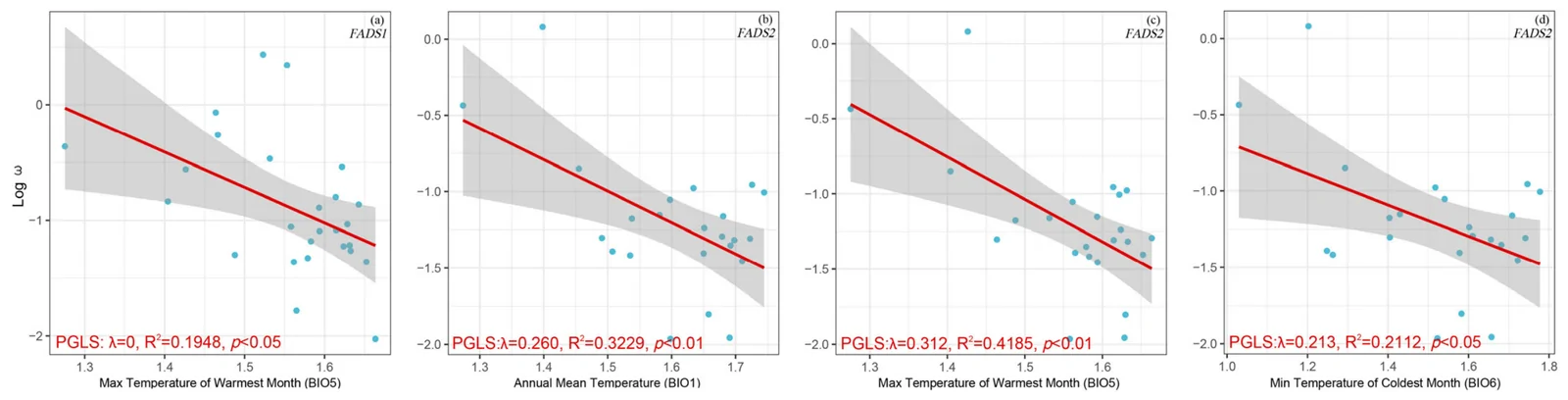
Regression analyses between ω values and climatic factors via PGLS. (**a**) Relationship between ω values of *FADS1* and BIO5. (**b**) Relationship between ω values of *FADS2* and BIO1. (**c**) Relationship between ω values of *FADS2* and BIO5. (**d**) Relationship between ω values of *FADS2* and BIO6. Solid lines represent regressions from PGLS methods with slope and intercept values displayed.

We further conducted phylogenetic generalized least squares (PGLS) analyses to examine the relationship between the climatic factors and the evolutionary rates of *FADS* genes. The regression results indicated a significant negative correlation between the ω values of *FADS1* and BIO5 (Figure 4a, Table 1). Additionally, the ω values of *FADS2* exhibited significant negative correlations not only with BIO1 but also with BIO5 and BIO6 (Figure 4, Table 1). The λ values of the phylogenetic signals estimated in the PGLS models were relatively low (0–0.312), suggesting that the variation in the ω values of the *FADS* genes is weakly structured by phylogeny and more strongly associated with environmental variables. These findings imply that lower temperatures in the habitats of rodents are associated with higher ω values of *FADS1* and *FADS2*.

### 3.5. Branch Model Analyses Across Climatic Regimes and Different Lineages

Branch models (two-ratio models) were employed to further determine whether elevated evolutionary rates are specific to environmental temperature, with the Low, Medium, and High groups designated as foreground branches to assess the selection pressure across the different factor groups. When the Low groups for BIO1, BIO5, and BIO6 were analyzed as foreground branches, all two-ratio models for *FADS1* were significantly superior to the null models (*p* < 0.05), with the foreground ω1 values consistently higher than the background ω0 values (Table 2). The ω value for the Low group of BIO5 was the highest. Similarly, the analysis of *FADS2* revealed a significant increase in the ω values within the Low group of BIO5 (ω1 = 0.1094; ω0 = 0.0637). Conversely, when the Medium and High groups were designated as foreground branches, certain two-ratio models also exhibited a significant enhancement in performance compared to the null model. However, the ω values for these foreground branches were lower than those for the background values, suggesting relatively stronger purifying selection in these lineages.

According to the aforementioned results, the Low group of BIO5 exhibits the strongest signal of accelerated evolution. To further validate this pattern, we selected five species with the lowest BIO5 values (*Dicrostonyx torquatus*, *Marmota himalayana*, *Urocitellus parryii*, *Alexandromys oeconomus*, and *Clethrionomys rutilus*), all of which are adapted to extremely cold environments. Additionally, other cold-adapted rodents inhabiting similarly frigid habitats were also included as foreground branches, with High group species used as background. The results showed that the two-ratio model was significantly different from the null model. For *FADS1*, ω was 0.3901 in the low-temperature species versus 0.0847 in the background, and for *FADS2*, ω was 0.1409 versus 0.0683 in the background (Table 2). These results further support the association between extreme cold environments and the accelerated evolution of *FADS* genes. To determine whether this pattern is general across a broader range of mammals, additional analyses were performed using cold-adapted species from artiodactyls, carnivores, and lagomorphs. The results from the branch model indicated that the ω values in the foreground branches for *FADS1* and *FADS2* were 0.3049 and 0.1328, respectively, both higher than the background values (0.0938 and 0.0756), suggesting that the cold-associated acceleration of *FADS* evolution may represent a convergent pattern across mammals.

### 3.6. Branch-Site Analyses Reveal Episodic Positive Selection in Cold-Associated Lineages

Among the tested cold-associated lineages, significant branch-site signals were detected in the *FADS1* gene of *S. dauricus* and *M. oregoni*, and BEB analysis identified six and twelve candidate positively selected sites in these two lineages, respectively (Table S5). The identified sites showed high posterior probabilities (>0.95), including six strongly supported sites in *S. dauricus* (PP = 0.954–0.999) and twelve sites in *M. oregoni* (PP = 0.972–0.991). These results indicate that episodic positive selection may have affected specific codons in some cold-associated lineages, despite the overall predominance of purifying selection across the *FADS1* gene. Together with the branch model analyses, these findings support temperature-associated variation in selective pressures rather than widespread positive selection driven by cold environments.

## 4. Discussion

In this study, we integrated phylogenetic comparison methods, molecular evolutionary analyses, and climatic datasets to investigate the effect of temperature on the evolutionary rates of the *FADS1* and *FADS2* genes in rodents. The results from multiple analyses consistently demonstrated that species inhabiting lower-temperature environments exhibited significantly higher ω values and elevated lineage-specific evolutionary rate estimates compared with species in warmer environments. This pattern was also detected in expanded mammalian datasets, suggesting that the observed trend is not restricted to rodents but may occur across multiple mammalian lineages. These results are consistent with evidence from human population genetic studies showing that *FADS* loci can be subject to natural selection [36,37]. Although most lineage-specific ω values remained below 1, the elevated ω values observed here indicate temperature-associated variation in selective pressures, which may reflect changes in the purifying selection strength and, in some lineages, episodic positive selection acting on specific codons.

This study quantitatively evaluated the impact of temperature on evolutionary rates using phylogenetic analysis of variance (phyANOVA) and phylogenetic generalized least squares (PGLS). The findings consistently showed that lower temperatures, specifically Max Temperature of Warmest Month (BIO5) and Annual Mean Temperature (BIO1), were associated with higher ω values of *FADS* genes. All analyses presented above confirm a strong significant correlation between BIO5 and the ω values of *FADS* genes. As BIO5 represents the maximum thermal constraint of a given habitat, lower BIO5 values typically indicate reduced exposure to heat extremes and are commonly associated with persistently cold climatic regimes [56,57,58]. Accordingly, the observed association suggests that long-term exposure to colder climatic conditions may be related to altered selective pressures on *FADS* genes rather than uniformly stronger positive selection. This pattern is also evident in high-latitude and high-altitude regions, where sustained cold conditions appear to serve as an important selective force driving cold adaptation [59,60,61]. Molecular evolution analyses further confirmed that species within the low-temperature group experienced significantly accelerated evolutionary rates of *FADS* genes, whereas those in the Medium- and High-temperature groups were subject to stringent purifying selection. This pattern provides empirical support for the hypothesis that environmental factors can shape molecular evolution rates, as reflected by lineage-specific variation in nonsynonymous substitution rates [49,62,63,64].

We further performed branch-site analyses using individual low-temperature-associated lineages as foreground branches to determine whether the elevated lineage-level ω values were accompanied by positive selection acting on particular codons. Significant branch-site signals were detected in *Spermophilus dauricus* and *Microtus oregoni*: Bayes Empirical Bayes analyses identified six candidate positively selected sites in *S. dauricus* and twelve candidate sites in *M. oregoni*. These findings provide complementary evidence to the branch model analyses by showing that, although *FADS* genes remain predominantly under purifying selection at the gene-wide level, episodic positive selection may have acted on a subset of codons in particular cold-associated lineages. Thus, the elevated ω values observed in low-temperature species may reflect a combination of lineage-wide variation in selective constraint and site-specific selective episodes rather than pervasive positive selection across the entire gene.

The variation in the temperature-associated evolutionary rates of *FADS* genes has a plausible physiological and biophysical framework [12,13,14,23]. The homeoviscous adaptation (HVA) hypothesis posits that organisms exposed to low-temperature conditions maintain membrane fluidity homeostasis by increasing the proportion of PUFAs in their membrane lipids [9,15,16]. *FADS1* and *FADS2* encode key rate-limiting enzymes in the biosynthesis of long-chain PUFAs, which are essential components of membrane phospholipids [30,31]. The elevated evolutionary rates observed in *FADS* genes may therefore be related to lineage-specific modifications of lipid metabolic regulation under different thermal environments; however, the present comparative analyses do not demonstrate that these sequence changes directly modify enzyme function. Potential effects on substrate affinity, catalytic efficiency, or membrane lipid homeostasis under low-temperature conditions remain hypotheses requiring functional validation [39,65,66]. Furthermore, the low-temperature environment may impose additional selective pressure on *FADS* through functional pleiotropy. To regulate their body temperatures, rodents inhabiting cold environments depend significantly on non-shivering thermogenesis, which is mediated by brown adipose tissue (BAT) [18,23]. Long-chain PUFAs, catalyzed by FADS, serve as natural ligands for critical nuclear receptors involved in the thermogenesis pathway, such as PPARs [18,19,20]. Consequently, the simultaneous requirement to maintain membrane lipid homeostasis and support energy metabolism and heat production provides a biologically plausible framework through which thermal environments could influence the evolution of *FADS* genes.

Both *FADS1* and *FADS2* exhibited overall increases in ω values under cold conditions, yet they demonstrated distinct evolutionary characteristics. The signals detected in the low-temperature species were significantly stronger for *FADS1* than for *FADS2*. The ω value for the low-temperature group of BIO5 reached 0.3425, whereas the value for the background branches was only 0.0796 (Table 2). In the expanded cold-adapted rodent dataset, this value further increased to 0.3901, with the background branch at 0.0847. In contrast, the overall ω value of the *FADS2* gene remained relatively low. This finding suggests that the increased evolutionary rate more likely reflects a moderate relaxation of purifying selection, which may allow its expression products to be optimized on the basis of the original function to enhance the efficiency of unsaturated fatty acid synthesis [31,67]. This evolutionary pattern is consistent with the characteristics of function-specific genes, wherein core functions are preserved through purifying selection, while the efficiency of optimizing functions is improved through moderate increases in evolutionary rates [1,68].

This divergence in evolutionary patterns can be attributed to the functional distinctions between the two enzymes in the regulation of long-chain PUFA synthesis. FADS2 (Δ6-desaturase) is located at the entry point of the PUFA biosynthesis pathway, where it catalyzes the first desaturation step of linoleic acid and α-linolenic acid [31]. In addition to its canonical Δ6-desaturase activity, FADS2 has been reported to exhibit alternative desaturase activities, including Δ8-desaturation in certain species, indicating a degree of substrate flexibility [69]. As an upstream enzyme in the long-chain polyunsaturated fatty acid biosynthetic pathway, *FADS2* is likely subject to relatively strong purifying selection to maintain metabolic flux stability [70,71]. In contrast, FADS1 (Δ5-desaturase) is positioned downstream in the pathway and is directly responsible for synthesizing crucial terminal effector molecules, such as arachidonic acid and eicosapentaenoic acid [30,31]. As a functionally specific enzyme operating at a terminal-branch point of lipid biosynthesis, *FADS1* may be subject to lineage-specific selective pressures associated with metabolic demands, which may facilitate the adaptive fine-tuning of catalytic properties under environmental constraints [1]. The elevated evolutionary rate identified in this study may influence changes in enzyme–substrate affinity or the conformational flexibility of proteins at low temperatures. Consequently, *FADS1* may serve as a candidate gene potentially involved in thermal-associated lipid metabolic evolution in mammals exposed to cold conditions.

In the PGLS analyses, *FADS* genes exhibited relatively weak phylogenetic signals with extremely low λ values (0 for *FADS1* and 0.260–0.312 for *FADS2*, Table 1), indicating weak phylogenetic dependence on trait variation. This pattern suggests that interspecific variation in evolutionary rates is only partially structured by phylogeny and may be influenced by environmental factors such as temperature [72,73]. This result is consistent with the ecological characteristics of rodents, which typically exhibit limited dispersal ability and are therefore more likely to respond to environmental stress through local adaptation rather than long-distance migration [42]. This pattern suggests that ecological factors may play a stronger role than phylogenetic inertia in shaping variation in *FADS* evolutionary rates. Furthermore, when extending the analysis to additional mammalian lineages, including artiodactyls, carnivores and lagomorphs residing in extremely cold environments, a similar trend of accelerated evolution in *FADS* genes was observed. This consistency across multiple independent lineages aligns with the idea that lipid metabolism genes may be recurrent targets of selection under cold environmental conditions, suggesting a potentially convergent adaptive response among mammals exposed to extreme cold climates [64,74,75].

From the perspective of the trade-off between molecular evolutionary dynamics and thermodynamic stability, cold-adapted enzymes that function in low-temperature environments often maintain catalytic activity through increased structural flexibility. This adaptation is frequently associated with reduced thermal stability at higher temperatures, reflecting a general stability–activity trade-off in protein evolution [1,68,76]. The observed temperature-associated evolutionary variation in *FADS* genes in cold environments likely reflects the long-term optimization of lipid metabolism under low-temperature constraints. However, such temperature-specific specialization may also impose evolutionary constraints under rapid environmental warming, potentially leading to decreased metabolic performance and fitness in affected populations, particularly under scenarios of accelerated climate change [41,77,78]. In particular, rodents with narrow distribution ranges and strong microhabitat dependencies, such as those endemic to alpine or high-latitude regions, may exhibit increased vulnerability to environmental change due to limited dispersal ability and ecological specialization [79,80,81]. Consequently, if temperature-associated variation in lipid metabolic genes contributes to physiological specialization, then such specialization could potentially constrain responses to rapid climate warming. This study may provide a theoretical framework for evaluating the evolutionary potential and survival risks of species amid global warming.

## 5. Conclusions

This study integrated phylogenetic comparative methods, molecular evolutionary analyses, and climatic datasets to investigate temperature-associated evolutionary patterns of the *FADS1* and *FADS2* genes in rodents and other mammals. The results showed that species inhabiting colder environments generally exhibited higher ω values than species from warmer environments, indicating temperature-associated variation in evolutionary rates and selective constraints among lineages. Branch model analyses also supported heterogeneous selective pressures between low-temperature and warmer-temperature lineages, and similar patterns were observed in expanded mammalian datasets. These findings suggest that environmental temperature may be an important ecological factor associated with variation in the selective pressures on and evolutionary trajectories of *FADS* genes across mammals. Given the essential role of *FADS1* and *FADS2* in long-chain polyunsaturated fatty acid biosynthesis, the observed evolutionary variation may be related to differences in lipid metabolic regulation under contrasting thermal environments. Overall, this study provides new insights into the molecular evolution of lipid metabolism genes in wild mammals and contributes to a better understanding of how climatic factors may shape evolutionary variation in metabolic genes across diverse mammalian lineages.

## Figures and Tables

**Table 1:** Significant results of PGLS analyses for ω values with climatic factors.

Response Variable	Predictor Variable	Slope	λ	R^2^	*p*
*FADS1*	Annual Mean Temperature (BIO1)	−1.5811	0.144	0.0696	0.1032
*FADS1*	Max Temperature of Warmest Month (BIO5)	−3.0543	0	0.1948	0.0139 *
*FADS1*	Min Temperature of Coldest Month (BIO6)	−0.5047	0.428	−0.0039	0.3515
*FADS* *2*	Annual Mean Temperature (BIO1)	−2.1872	0.260	0.3229	0.0028 **
*FADS* *2*	Max Temperature of Warmest Month (BIO5)	−3.1511	0.312	0.4185	0.0005 **
*FADS* *2*	Min Temperature of Coldest Month (BIO6)	−1.0698	0.213	0.2112	0.0158 *

* *p* < 0.05; ** *p* < 0.01.

**Table 2:** Results of branch model analyses on *FADS* genes.

Dataset	Gene	Partition	Foreground	Model	lnL	−2lnΔL	*p*	Parameter Estimates
Rodentia (27 species)	*FADS1*			One-ratio model	−7041.85			
		BIO1	Low	Two-ratio model	−7038.93	5.86	0.02	ω1 = 0.1435, ω0 = 0.0951
		BIO1	Medium	Two-ratio model	−7040.93	1.86	0.17	
		BIO1	High	Two-ratio model	−7040.84	2.02	0.16	
		BIO5	Low	Two-ratio model	−7009.91	63.89	0.00	ω1 = 0.3425, ω0 = 0.0796
		BIO5	Medium	Two-ratio model	−7038.53	6.66	0.01	ω1 = 0.0734, ω0 = 0.1143
		BIO5	High	Two-ratio model	−7039.53	4.65	0.03	ω1 = 0.0767, ω0 = 0.1125
		BIO6	Low	Two-ratio model	−7038.93	5.86	0.02	ω1 = 0.1435, ω0 = 0.0951
		BIO6	Medium	Two-ratio model	−7040.93	1.86	0.17	
		BIO6	High	Two-ratio model	−7040.84	2.02	0.16	
	*FADS2*			One-ratio model	−7187.70			
		BIO1	Low	Two-ratio model	−7187.25	0.91	0.34	
		BIO1	Medium	Two-ratio model	−7185.45	4.50	0.03	ω1 = 0.0467, ω0 = 0.0734
		BIO1	High	Two-ratio model	−7187.09	1.23	0.27	
		BIO5	Low	Two-ratio model	−7184.03	7.35	0.01	ω1 = 0.1094, ω0 = 0.0637
		BIO5	Medium	Two-ratio model	−7185.88	3.65	0.06	
		BIO5	High	Two-ratio model	−7184.75	5.92	0.02	ω1 = 0.0475, ω0 = 0.0753
		BIO6	Low	Two-ratio model	−7187.25	0.91	0.34	
		BIO6	Medium	Two-ratio model	−7185.45	4.50	0.03	ω1 = 0.0467, ω0 = 0.0734
		BIO6	High	Two-ratio model	−7187.09	1.23	0.27	
Rodentia (18 species)	*FADS1*			One-ratio model	−6148.17	76.27	0.00	ω1 = 0.3901, ω0 = 0.0847
				Two-ratio model	−6110.04			
	*FADS* *2*			One-ratio model	−5472.05	14.35	0.00	ω1 = 0.1409, ω0 = 0.0683
				Two-ratio model	−5464.88			
Rodentia + Lagomorpha + Carnivora + Artiodactyla (27 species)	*FADS1*			One-ratio model	−6656.04	54.23	0.00	ω1 = 0.3049, ω0 = 0.0938
				Two-ratio model	−6628.93			
	*FADS* *2*			One-ratio model	−6522.45	9.79	0.00	ω1 = 0.1328, ω0 = 0.0756
				Two-ratio model	−6517.56			

## Data Availability

The data presented in this study are available upon reasonable request from the corresponding author.

## Supplementary Materials

The following supporting information can be downloaded at: https://www.mdpi.com/article/10.3390/ani16162600/s1, Table S1: Species, gene accession number or scaffold information for *FADS* genes in this study; Table S2: Climate datasets; Table S3: Likelihood ratio tests of branch models for the *FADS* genes; Table S4: The results of phyANOVA analysis; Table S5: Likelihood ratio tests of branch-site models for the *FADS* genes.
